# Revealing the complex conduction heat transfer mechanism of nanofluids

**DOI:** 10.1186/s11671-015-0954-8

**Published:** 2015-06-03

**Authors:** A Sergis, Y Hardalupas

**Affiliations:** The Department of Mechanical Engineering, Imperial College London, London, SW7 2AZ UK

**Keywords:** Nanofluid, Nanoparticle, MDS, Heat transfer

## Abstract

Nanofluids are two-phase mixtures consisting of small percentages of nanoparticles (sub 1–10 %vol) inside a carrier fluid. The typical size of nanoparticles is less than 100 nm. These fluids have been exhibiting experimentally a significant increase of thermal performance compared to the corresponding carrier fluids, which cannot be explained using the classical thermodynamic theory. This study deciphers the thermal heat transfer mechanism for the conductive heat transfer mode via a molecular dynamics simulation code. The current findings are the first of their kind and conflict with the proposed theories for heat transfer propagation through micron-sized slurries and pure matter. The authors provide evidence of a complex new type of heat transfer mechanism, which explains the observed abnormal heat transfer augmentation. The new mechanism appears to unite a number of popular speculations for the thermal heat transfer mechanism employed by nanofluids as predicted by the majority of the researchers of the field into a single one. The constituents of the increased diffusivity of the nanoparticle can be attributed to mismatching of the local temperature profiles between parts of the surface of the solid and the fluid resulting in increased local thermophoretic effects. These effects affect the region surrounding the solid manifesting interfacial layer phenomena (Kapitza resistance). In this region, the activity of the fluid and the interactions between the fluid and the nanoparticle are elevated. Isotropic increased nanoparticle mobility is manifested as enhanced Brownian motion and diffusion effects

## Background

Nanofluids are binary mixtures containing solid nanoparticles (usually below 100 nm in size) and a carrier/base fluid (usually conventional coolants) invented by U.S. Choi during the 1990s [1]. The concentration of nanoparticles in the mixture is of the order of sub 1 %vol up to 10 %vol. Despite the small concentration of nanoparticles and their small size, it has been proven experimentally that the measured thermal performance exceeds the thermal enhancement predicted by classical thermodynamic models. Nanofluids exhibit an enhancement over their base fluids of the order of 5–9 %, 10–14 %, 40–44 % and 100–200 %, respectively, for the purely conductive, mixed convective/conductive, pool boiling and critical heat flux (CHF) heat transfer modes. The physical mechanisms that give rise to this enhancement are not yet understood, and hence, the design flexibility offered by the increased degrees of freedom of the mixture parameters (nanoparticle and carrier combination materials, flow application type and state, nanoparticle size, surface treatment, shape and concentration as well as temperature range of application) for maximising their performance at specific applications cannot be defined [2–12].

Nanofluids have recently attracted attention for several other applications which expand even further their capabilities as working fluids. Recent work was published on nanofluids in peristaltic flows and porous channels [13–15]. These applications are of high importance for biology and biomedicine that will help understand several physical processes which are yet unknown. Several other applications considered the flow of non-Newtonian nanofluids in the presence of magnetohydrodynamics, radiation and porous media to derive analytic solutions to describe them. This has a great importance for several industrial applications we are currently using but have not yet streamlined and fully understood or are future candidates for nanofluid applications, for example, the flow of liquid metals, cosmic plasmas, MHD pumps and power generators, electrostatic precipitation processes, the petroleum industry, purification of crude oil and several others [13, 14, 16–21]. Finally, the research community also started to include studies on the natural and forced convection of nanofluids in porous media which is currently a rare novelty in the field [22–24]. All of the new studies are trying to correlate the thermal performance of nanofluids to the types of nanoparticles and base fluids these are suspended into, the nanoparticle concentration, size, material and shape as well as the pressure for pumping these fluids under certain conditions around a circuit. The purpose of this novel work is to either evaluate the applicability of nanofluids to current or future industrial processes or comprehend current industrial, biological and physical processes.

The current study is a continuation of an earlier investigation [25] to build and run a large-scale deployment of a molecular dynamics simulation (MDS) code and to simulate from first principles and with minimal assumptions a novel single particle nanofluid to study the conductive heat transfer mechanisms employed. The analysis is achieved by comparing the nanoparticle trajectories across thousands of simulations and iterations with those of a carrier fluid molecule to quantify the effects of thermal diffusivity. All quantities presented in this work are non-dimensional unless stated otherwise.

## Methods

### Code development

The standard 6–12 Lennard Jones model (Eq. 1) is used to depict the interatomic potential energy in between a pair of liquid argon atoms in a 2D domain and is based on [26, 27] while the required dynamic quantities are obtained using the leapfrog integration method.

1 $$ \boldsymbol{u}\left({\boldsymbol{r}}_{ij}\right)=4\varepsilon \left[{\left(\frac{\sigma }{{\boldsymbol{r}}_{ij}}\right)}^{12}-{\left(\frac{\sigma }{{\boldsymbol{r}}_{ij}}\right)}^6\right]+\varepsilon, \kern1em \left|{\boldsymbol{r}}_{ij}\right|\le {r}_c={2}^{\frac{1}{6}}\sigma $$

where, ***r***_*ij*_ is the distance vector between the *i*th and *j*th pair of atoms, ***u***(***r***_*ij*_) is the potential energy vector between the *i*th and *j*th pair of atoms, *ε* is the strength of interaction, *σ* is the characteristic length scale and *r*_c_ is the cut-off distance at which we assume that the attractive tail of the model is no longer significant.

The quantities are non-dimensionalised by using *r* with *rσ* for the units of length, *e* with *eε* for the units of energy and *t* with $t\sqrt{m{\sigma}^2/\varepsilon }$ for the units of time. The system is initialised at a temperature of 1 non-dimensional (ND) unit and a number density of 0.8 ND units in a square domain size. The origin of the *x*-*y* coordinate system used lies at the geometrical centre of the domain. The code reaches steady state after 5000 iteration steps (the equivalent of 25 ND time units). Each simulation lasts 1e5 iterations, which is the equivalent of 500 ND time units, while each test case (baseline fluid and nanoparticle formation tests) contained about 1500 realisations to obtain appropriate statistics.

Periodic walls are in place in the horizontal *x*-direction while adiabatic stochastic boundaries are in place in the vertical *y*-direction. The domain is large enough to prevent wraparound effects over the periodic boundaries. A temperature gradient is established in the domain by keeping the lower wall at a constant ND temperature of 1 unit, while varying the temperature of the top wall by more than 1 ND temperature unit. The hard stochastic wall model introduces a system energy leakage which is counteracted by elevating the upper wall temperature by at least 0.16 ND temperature units over the lower cold wall temperature. A linear temperature gradient naturally evolves under these parameters. For the cases tested, the temperature gradient is established after a maximum of 25,000–30,000 iterations (the equivalent of 100–150 ND time units).

**Fig. 1:**
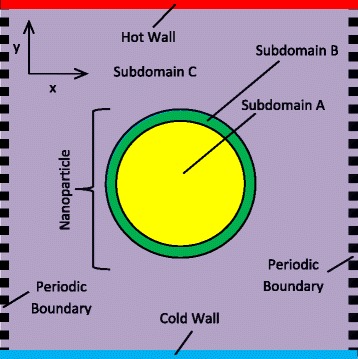
Domain set-up schematic indicating main features

The nanoparticle is formed by binding argon atoms together via the creation of three subdomains as shown in Fig. 1 [27]. Inside subdomain A, a crystallic structure is enforced with the atoms in that area interacting with an *ε* equal to 5 ND units. Subdomain B is used to prevent atomic evaporation from the surface of the nanoparticle, and hence, the strength of interaction reduces to 2 ND units, while in subdomain C, the liquid argon molecules exist with an interatomic interaction strength of 1 ND unit. The effective *ε* used in Eq. 1 for the paired interactions from different subdomains is calculated by multiplying the individual interactions of each atom according to its location. For all cases, the nanoparticle and baseline fluid atoms are released from the middle of the domain in a constant linear temperature gradient of 0.02 ND temperature gradient units.

The model was benchmarked via three tests widely used in the field. The first compared the randomness function of the system before and after the developed routines were applied (excluding the nanoparticle formation routine) to detect any possible changes in the distribution of atoms in the domain. The second test involved plotting the radial distribution functions (RDF) of the system before and after a nanoparticle was formed. The third and final test involved plotting the normalised velocity autocorrelation function (VACF) of a nanoparticle and a normal argon fluid atom (more information can be found in an earlier study performed [25]). All three tests indicated good agreement with the literature [26–35].

The code is composed on Fortran 90 and compiled for 64-bit Windows 7 machines via Microsoft Visual Studio 2008. The code is deployed through a 4000 core HTCondor cluster installed at the South Kensington Campus of Imperial College London. Developing the code and obtaining the results took about eight million CPU hours. More information regarding the methodology involved in composing, running and developing the code and the HTCondor system can be obtained from [25].

### Results post processing

Large numbers of simulations are necessary to be able to form the statistics of the macroscopic system behaviour from the molecular level. Post processing and visualisation of the data is performed through Matlab since it provides the required flexibility. Compression routines were devised to handle the large volume of data recorded based on recursive equations to quantify the statistics of the system. More information regarding the compression routines can be obtained from [25].

A size parametric test was conducted involving four nanoparticle sizes—0.6, 1.1, 2 and 2.9 nm in diameter. The domain size scales linearly with the diameter of the nanoparticle to ensure that the effect of the domain size on the results is minimised as well as preserving computational time efficiency. The minimum domain size was kept at 1600 atoms, and it was valid for the lowest two nanoparticle sizes tested.

It was decided to perform the following studies to quantify the results obtained from the solutions. An average distance vector study quantifies the 1D average trajectory of the nanoparticle and baseline fluid cases. The dimension of interest is along the temperature gradient. This study will indicate the average behaviour of the nanoparticles and fluid atoms in the domain. 3D histogram studies on the one-dimensional penetration distance covered by the particles and fluid atoms as a probability density function difference quantify the extent of penetration along the temperature gradient. 2D spread plot studies quantify the penetration distances covered by the nanoparticles and fluid atoms in the domain as probability density function differences in both dimensions used. The penetration distance is correlated with the heat diffusion distance. Absolute distance-covered plot studies indicate the overall activity of the nanoparticle and baseline fluid atoms. Residence time plots show the average percentage of residence time of the nanoparticle and fluid atoms inside the hot and cold parts of the domain. The self-diffusion coefficient studies (Eq. 2) indicate the self-diffusivity of nanoparticles and fluid atoms across the simulation. The factor correlates with thermal diffusion and provides a means of understanding the effects of nanoparticles on the conductive thermal heat transfer mode.

2 $$ {D}_{\mathrm{self}}=\frac{1}{2}{\displaystyle {\int}_{t_1}^{t_2}\Big\langle \boldsymbol{V}\left({t}_1\right)\cdot \boldsymbol{V}\left({t}_1+t\hbox{'}\right)dt\hbox{'}} $$

where *D*_self_ is the self-diffusion coefficient, *t*_1_,*t*_2_ are the reference times where the diffusivity investigation is initiated (*t*_1_) and ended (*t*_2_) in ND time units, *t*′ is the integral time variable and ***V*** is the particle/fluid atom velocity vector in ND velocity units.

Finally, a temperature spatial distribution study was performed to quantify local temperature effects around and inside the nanoparticles.

## Results and Discussion

### MDS results

**Fig. 2:**
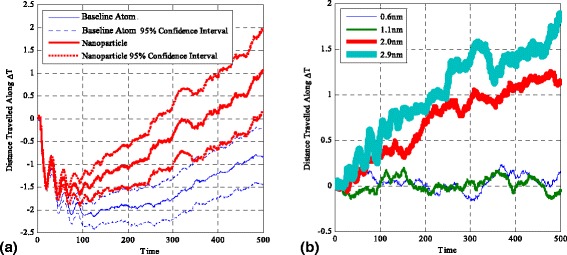
**a** The average distance vector, travelled along the direction of the temperature gradient, for the 2.9-nm particle and baseline atom. **b** The parametric study results on all sizes tested and the differences in distance covered by the nanoparticle and normal fluid atom

Figure 2a shows a comparison plot of the average distance vector travelled along the direction of the temperature gradient between a 2.9-nm nanoparticle and the baseline fluid atom. Figure 2b shows the results of the difference in the distance vector between a nanoparticle and the baseline fluid atom for different nanoparticle sizes. Figure 2a and the derived Fig. 2b contain distinctive features. On average, both the nanoparticle and baseline fluid atom display an initial sharp movement towards the cold region of the domain (‘kick’) followed by a slow recovery towards the hot region. For the entire duration of the ‘kick’, the nanoparticles lack behind the baseline fluid atom, while for the recovery, the nanoparticles are ahead of the baseline fluid atom. Additionally, for both cases, there seems to exist an oscillatory behaviour for times up to 100–150 time units, which corresponds to the system heating-up duration.

The initial ‘kick’ can be accounted for by thermophoresis. Thermophoresis is a force which acts to cause migration of particles and baseline fluid atoms against the temperature gradient of their surroundings according to Eq. 3 (macroscopically and ignoring temperature variations inside the nanoparticle itself) [36–38] and is hence responsible for causing an initial average motion of particles and baseline fluid atoms towards the cold region of the domain. The nanoparticle lacks behind the baseline fluid atom due to its large mass; hence, inertia effects come into place to reduce the effects of thermophoresis on the followed trajectory.

3 $$ {F}_{\mathrm{TP}}\propto -\frac{d_{\mathrm{p}}\nabla T}{T} $$

where *F*_TP_ is the thermophoresis force, *d*_p_ is the particle/atomic diameter and *T* is the temperature of the domain.

The thermophoresis is initially dominant as the local ∇*T* is large during the system heat-up period while *T* is small. When the system reaches a constant global average temperature, the thermophoretic effects reduce as the local ∇*T* reduces to the global one and the average temperature of the system increases. The thermophoresis causes a global atomic number density increase towards the cold region of the domain. The formed number density gradient has an opposite sign to the temperature gradient established in the domain. According to Fick’s first law of diffusion (Eq. 4), a diffusion force will be established, which acts in the opposite direction as the number density gradient initially established. This force becomes dominant over the thermophoretic force and hence is responsible for the slow recovery—evident for both the nanoparticle and baseline—back to the hot side of the domain.

4 $$ J=-D\nabla \varPhi $$

where *J* is the diffusion flux, *D* is the diffusion factor/coefficient and *Φ* is the concentration of particles/fluid atoms in consideration. However, this cannot still explain the increased distance covered by the nanoparticle compared to the normal fluid atom and how this correlates with the size of the nanoparticle.

The initialisation of the simulation and the application of the hot wall on a ‘cold’ system induce a large perturbation, which then propagates through the system as a thermal wave. The perturbation dies out as the system reaches a constant global temperature as the local temperature gradients are smoothed out to the steady state ones. This has as a result the oscillatory behaviour of particles and fluid atoms seen for times up to 100–150 ND time units in Fig. 2. The effects are larger for larger dimension systems as by keeping a constant temperature gradient across the domain results in higher hot wall temperatures—hence, making the initial local perturbation larger. The increase of domain size has also a direct effect on the duration of oscillations in time as the larger perturbations require longer times to traverse and eventually die out in a larger domain upon reaching steady state.

**Fig. 3:**
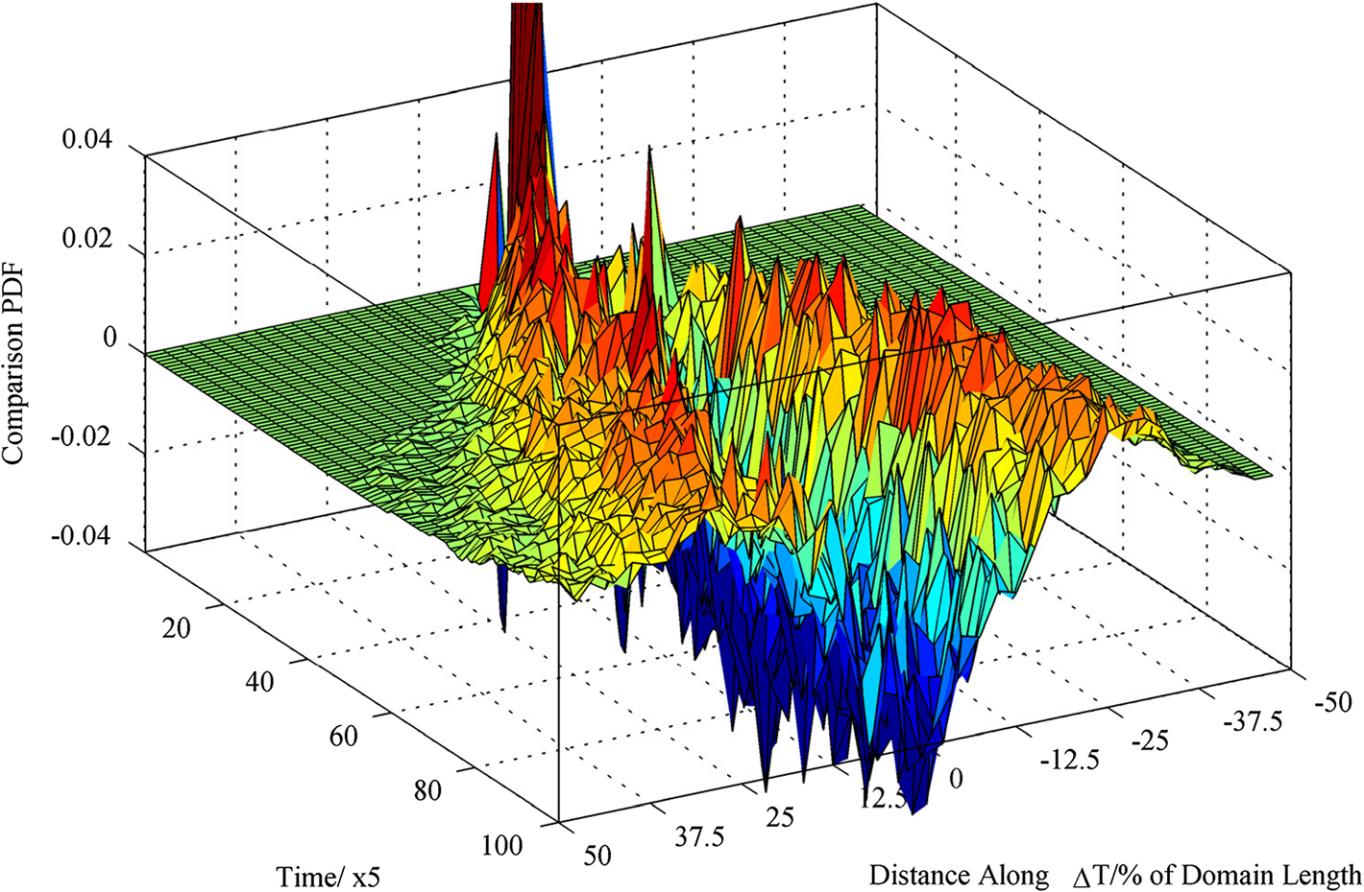
Comparison PDF of the one-dimensional spatial distribution between a 2.9-nm nanoparticle and the baseline atoms against time. Positive values indicate that the PDF of the nanoparticle is larger than the PDF of the baseline fluid atom. *PDF* probability density function

Focusing on the one-dimensional spread of particles and fluid atoms along the temperature gradient, Fig. 3 shows an example comparison plot of the one-dimensional spatial distribution between a 2.9-nm nanoparticle and the baseline fluid atom against time (positive peaks indicate an increased probability for a nanoparticle to occupy the investigated position while negative peaks indicate that the baseline fluid atom is most probable to occupy the investigated position). The 3D histograms quantified the effects of the temperature gradient and confirmed that there is an increased probability that the nanoparticles will travel further away from their initial release point compared to the baseline. The effect is more pronounced as the nanoparticle size is increased. The comparison produces a noisy profile near the hot wall as the probability of differential particle and fluid atom distribution in this more active region is less correlated (hence, the wrinkling of the zero-difference sheet closer to the hot wall in Fig. 3).

Nanoparticle and baseline fluid atom spread studies were also performed indicating that in two dimensions the nanoparticles have larger probabilities to travel further away from their release point compared to the baseline fluid atom. The larger spread of nanoparticles appeared isotropic, which indicated that the mechanism employed gives rise to an increased mobility in all directions, including those that do not contain any ‘driving’ temperature gradient (horizontal direction in this study).

**Fig. 4:**
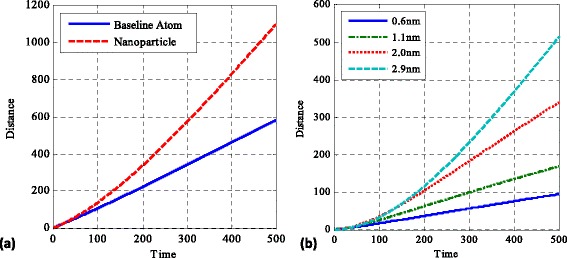
Absolute distance-covered plots for a 2.9-nm particle and the baseline fluid atom (**a**) and a size parametric study of the difference in the absolute distances covered between the nanoparticle and the baseline atom (**b**)

The absolute distance covered showed the activity of the nanoparticle and baseline fluid atom. Figure 4a shows an example plot for the 2.9-nm case, while Fig. 4b shows a size parametric study plot indicating the differences found between the absolute distance covered by a nanoparticle and the baseline fluid atom. The plots indicate significant differences with the nanoparticle covering up to twice the distance of a baseline fluid atom for the same time interval. The effect is more pronounced as the nanoparticle size is increased. Localisation studies were performed to quantify the residence time of the nanoparticles and base fluid atoms in either the cold or hot part of the domain (the two parts are defined geometrically in the direction of the temperature gradient. Half of the domain containing the hot wall is defined as the hot part while the remaining half of the domain containing the cold wall is defined as the cold part). The residence time studies performed indicate that even though overall the baseline fluid atom and nanoparticles spend most of their time in the cold region (as their effective velocities are lower in that region), the nanoparticle can spend up to 6.3 % more time inside the hot region compared to the baseline fluid atom. Most importantly, for longer system running times, there is a tendency for the residence time spent in either the hot or cold part of the domain to reach a 50–50 % distribution for the nanoparticles, something which is not evident for the fluid atoms.

**Fig. 5:**
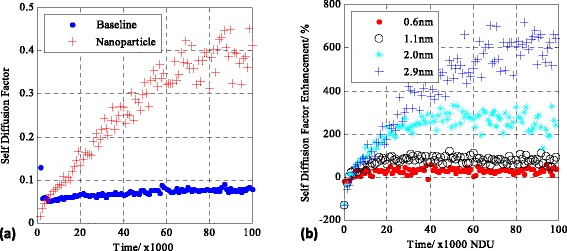
Self-diffusion coefficient for a 2.9-nm particle and the baseline (**a**) and a size parametric study comparison of the difference in the self-diffusion coefficients between nanoparticles and the baseline (**b**)

The latter results on the increased activity of nanoparticles are also confirmed by calculating the self-diffusion coefficient for the nanoparticle and baseline fluid atom (Fig. 5). Figure 5a shows the self-diffusion coefficient for a 2-nm nanoparticle and the corresponding baseline fluid atom, while Fig. 5b shows a comparison on the parametric size investigation of the self-diffusion coefficients between a nanoparticle and the baseline fluid atom. As expected, the baseline fluid atom has a fixed self-diffusion coefficient throughout the simulation with an insignificant increase towards longer computational times. The nanoparticle indicates a transient behaviour, reaching its maximum self-diffusion coefficient value after a significant amount of time. The effect is more profound for larger nanoparticles; particularly, for the 2.9-nm nanoparticle, the duration of the simulation is not enough for the nanoparticle to reach its maximum value. This is a clear indication that the nanoparticle is dramatically more active compared to the baseline fluid atom, which goes against the classical theories for the associated mean squared displacement of large particles compared to small micron-sized particles [39].

**Fig. 6:**
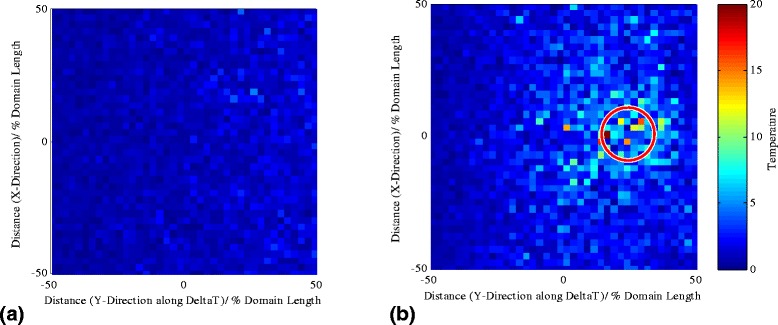
Instantaneous temperature distribution for the baseline fluid atom studies (**a**) and a 2.9-nm nanoparticle (**b**) in the domain. The temperature colour map has been preserved for both images. The red circle in (**b**) represents the nanoparticle crystallic boundaries

The local instantaneous temperature profiles (averaged over ten consecutive iterations which can be considered a very short time for considerable nanoparticle dynamic changes) of the baseline fluid atom and the 2.9-nm nanoparticle are plotted in Fig. 6 (the grid cell resolution for temperature calculations is 0.625 % of the domain length). The same domain with the nanoparticle assembly routines inactivated (Fig. 6a) and activated (Fig. 6b) is plotted. The nanoparticle appears to become a hotspot, which affects the surrounding area creating areas of mismatching surface temperatures between the nanoparticle and the surrounding fluid atoms.

### Formulation of a new type of heat transfer mechanism valid for nanofluids

There is strong evidence of the existence of a complex heat transfer mechanism employed by nanoparticles as indicated by the findings of the study. The nanoparticle, even though it is larger in size, appears to be more active compared to the smaller fluid atoms when placed in a temperature gradient. This goes against the classical thermodynamic theories for diffusing particles [39]. Previous investigations on the self-diffusion coefficient of nanoparticles in domains without the presence of a temperature gradient also indicated departure from Einstein’s diffusion relationship for micron-sized particles; however, they were in agreement with the inverse proportionality relationship of the self-diffusion coefficient with the particle radius [34]. In addition, other studies with hard sphere or/and assembled nanoparticles using similar assembly models as the current investigation but without the presence of a temperature gradient indicated an overall lower self-diffusion coefficient for nanoparticles [27] compared to fluid molecules/atoms. The literature is in direct disagreement with the findings of this study concerning the activity of nanoparticles in isothermal domains and excluding thermal quantum effects.

Given the results of the current analysis, the authors of this paper have concluded that the effects of increased nanoparticle activity observed are due to a complex mechanism arising from the mismatch of the temperature (kinetic energy ensemble) across parts of the surface of the nanoparticles and the surrounding fluid atoms. The temperature disparity induces a local thermophoretic force, which causes the enhanced mobility effects observed on the nanoparticle. The mismatch is the outcome of different heat transfer coefficients across the crystallic structure of the nanoparticle and the fluid as well as the ability of the nanoparticle to translate and rotate, which as a result, gives rise to a different collective atomic activity (local temperature) between certain regions of the particle and the surrounding fluid atoms (this is confirmed in Fig. 6). The increased nanoparticle temperature arises from the fluid to solid incompatibility of energy transfer at which the energy inside the nanoparticle (apart from part of it getting converted into potential energy and translational and rotational motion of the nanoparticle) is accumulated in the form of high-frequency and small-amplitude vibrations. On the contrary, the energy in the fluid domain is stored in the form of translational motion, while part of it is converted into potential in between the atomic bonds. The collective effect of this classical mechanical model is also causing an increased activity region surrounding the nanofluid known from the literature as the Kapitza resistance [3, 5, 40].

The nanoparticle becomes more active for larger sizes. This can be explained via the new proposed mechanism, since the nanoparticle size increase leads to increasing probability of a temperature mismatch to occur across certain parts of the nanoparticle. In addition, translational and rotational events enhance even further the mismatching phenomena. Although the size parametric study was limited to smaller nanoparticle sizes due to the limited available computational time, it is expected that the phenomenon will reach a maximum when the inertial effects of the nanoparticle will overcome the observed diffusive phenomena. This mechanism is in agreement with the experimental observations linking nanoparticle size and thermal conductivity of a nanofluid [2–12], and it might hence explain the heat transfer enhancement exhibited by nanofluids—excluding any quantum effects.

In essence, the proposed mechanism links several speculations regarding the various physical heat transfer mechanisms involved that give rise to the observed heat augmentation phenomena. Our earlier studies [3] indicated that most researches explain the augmentation observed in this type of study (conductive mode) via the enhancement of Brownian motion, the interfacial layer theory (Kapitza resistance) or a combination of enhanced Brownian motion and an aggregation and diffusion enhancement mechanism. Thermophoresis was also one of the speculated mechanisms found in the literature; however, it was not as popular as the former ones mentioned. The proposed new theory appears to unify popular speculated mechanisms into a single one. The temperature mismatching gives rise to local thermophoretic effects which increase the mobility of both the nanoparticle and the surrounding fluid atoms. The surrounding fluid interfacial layer of the nanoparticle also presents an increased activity known as the Kapitza resistance. The overall increased mobility observed results in enhanced Brownian motion and diffusion effects which in their turn give rise to the augmented heat transfer phenomena observed in nanofluids under the studied heat transfer mode. Aggregation phenomena cannot be verified, since a single nanoparticle study was considered.

## Conclusions

A MDS code was developed to simulate a single nanoparticle nanofluid with a temperature gradient to study, at a molecular level, the conductive heat transfer mechanisms involved. The code accuracy was successfully verified using a variety of MDS tests common to the field [25]. The results confirmed the presence of a complex heat transfer mechanism that gives rise to increased thermal diffusion phenomena, which intensify over the range of considered nanoparticle sizes. The observed trends are against the classical thermodynamic theories and reveal the presence of a new type of complex heat transfer mechanism. The new type of heat transfer mechanism justifies the experimental results found in the literature.

## Abbreviations

CHF: critical heat flux
MDS: molecular dynamics simulation
ND: non-dimensional
RDF: radial distribution function
VACF: velocity autocorrelation function
